# Role of Laser Powder Bed Fusion Process Factors in Determining the Porosity Formation in 3D Printing of Stainless Steel 316L: Theoretical Modeling and Experimental Verification

**DOI:** 10.3390/ma18245490

**Published:** 2025-12-05

**Authors:** Andrzej Stwora, Reza Teimouri, Jacek Habel

**Affiliations:** Faculty of Mechanical Engineering, Cracow University of Technology, 31-864 Cracow, Poland; andrzej.stwora@pk.edu.pl (A.S.); jacek.habel@pk.edu.pl (J.H.)

**Keywords:** additive manufacturing, laser powder bed fusion, modeling, porosity

## Abstract

In this study, an analytical model was developed to evaluate the influence of laser powder bed fusion (LPBF) process parameters on process-induced porosity during the 3D printing of stainless steel 316L. First, the temperature distribution, governed by a moving point heat source model of the laser, was used to predict the melt pool geometry during the melting stage. This prediction was then refined to account for the formation of the solidified cap. By analyzing the interaction between melt pool size and other process parameters, such as hatch spacing and layer thickness, criteria were established to distinguish between porosity caused by lack of fusion, porosity due to keyhole formation, and defect-free samples. A series of experiments were conducted, and porosity was measured using micro-CT analysis. The results showed that the porosity predicted by the model remained within an acceptable error range compared with the experimental measurements, with errors ranging from 10.5% to 24.78% and a mean error of 16.48%, demonstrating the accuracy of the developed model.

## 1. Introduction

Laser powder bed fusion (LPBF) is a widely used additive manufacturing technology that enables the fabrication of intricate, near-net-shape parts that are difficult to achieve with conventional subtractive methods. However, its industrial adoption faces challenges, because the mechanical properties of printed parts are highly sensitive to process parameters, which influence the formation of voids and defects. During printing, the material experiences a complex thermal history, which can lead to defects such as lack-of-fusion (LOF) pores, caused by insufficient melting due to low energy density, and gas porosity, generated during rapid solidification at high energy densities [1]. Therefore, controlling porosity through careful process parameter selection is crucial, requiring a safe balance in energy density.

Recent studies on porosity analysis and prediction in additive manufacturing increasingly rely on data-driven and physics-informed approaches. Smoqi et al. [2] developed a physics-informed model integrating melt pool signatures with machine learning to monitor and predict porosity formation during LPBF, achieving very high classification accuracy. A deep-learning framework demonstrated that layer-wise porosity can be predicted from the thermal histories of prior layers, validated via CT scans [3]. More recently, Atwya and Panoutsos introduced a prior-guided neural network using spectral emissions to localize micro-porosities in situ in LPBF, enhancing detection sensitivity while staying consistent with underlying process physics [4]. In addition, recent work on process monitoring and machine learning for defect detection in laser-based metal AM has highlighted how multi-modal sensing, such as optical, thermal, and acoustic signals, combined with ML classifiers can successfully identify porosity-related defects and distinguish between stable and unstable melt pool states [5]. However, despite these advances, machine learning remains extremely data hungry, often functions as a black box, and may not reveal the true underlying physical mechanisms driving porosity formation, limiting its interpretability and trustworthiness in scientifically and industrially critical applications.

Several studies have used experimental and numerical approaches to optimize LPBF by targeting process-induced porosity. Liu et al. [6] developed a computational fluid dynamics (CFD) model to assess the role of energy density in pore formation and defined a process window for producing pore-free samples. Vastola et al. [7] highlighted that bubble formation at high energy densities, due to trapped gas, is a key contributor to porosity in Ti-6Al-4V. Bakhtiarian et al. [8] experimentally studied the effect of LPBF parameters on the density and hardness of stainless steel 316L, revealing that hardness depends not only on microstructure size but also on process-induced porosity. Promoppatum [9] developed a numerical model to predict LOF in Ti-6Al-4V, which agreed with experiments but did not account for porosity formed at high energy densities. Wang et al. [10] and Ning et al. [11] proposed analytical models linking laser power and scan velocity to LOF porosity, but neglecting melt pool cap formation led to prediction errors. Ning et al. [12] later included powder size distribution, improving LOF prediction accuracy. Mukherjee and DebRoy [13] showed that porosity can be reduced by increasing energy density or decreasing hatch spacing and layer thickness, while Tang et al. [14] combined numerical simulations and analytical formulations to estimate LOF. Also, they have developed a numerical simulation model based on CFD analysis to predict the KH formation in LPBF of stainless steel [15].

For keyhole porosity, Wang and Liang [16] developed an analytical model validated with a limited number of samples, while King et al. [17] proposed an empirical–analytical approach, showing that a keyhole forms when the normalized enthalpy exceeds six.

Despite these contributions, most predictive models rely on time-consuming numerical simulations, while existing analytical models often neglect critical melt pool features, limiting accuracy. Many models focus on specific energy density regimes, failing to cover both LOF and keyhole porosity. Consequently, a major challenge in LPBF process design is the lack of fast, accurate, and broadly applicable predictive models. Empirical models are costly and material-specific, and FEM-based numerical models are computationally intensive.

To provide a clearer overview of previous studies on porosity modeling in the LPBF process, Table 1 summarizes their key features and identifies the research gaps that motivated the present study. In this context, an integrated, experimentally validated, multi-physics analytical framework offers an efficient approach to optimize LPBF parameters and to better understand their influence on porosity formation. Accordingly, this work aims to predict porosity induced by both lack-of-fusion (LOF) and keyhole mechanisms through the development of an analytical model that accounts for variations in laser power, scan velocity, hatch spacing, and layer thickness.

Unlike previous studies that primarily focused on a limited set of parameters or a single porosity formation mechanism, the present work investigates a broader parameter range encompassing multiple mechanisms. Moreover, the existing literature lacks a comprehensive parametric analysis that systematically examines the effects of different process factors on porosity evolution, a gap that this study addresses. To bridge this gap, the proposed framework not only establishes an analytical model but also performs a detailed parametric investigation to elucidate how various process parameters govern porosity development in the LPBF process, which represents a central contribution of this research.

It should be noted that the present study does not aim to characterize the detailed morphology of pores but to establish a predictive link between key LPBF parameters and the total porosity level. The total porosity is adopted as a representative indicator of defect content and is used to design a process window that ensures nearly porosity-free conditions (porosity < 0.5%). Since the porosity formation is a chaotic and highly nonlinear process, the detailed prediction of porosity descriptors such as size, shape, and tortuosity remains beyond the scope of this work and will be addressed as part of the future research framework.

## 2. Development Theoretical Framework

### 2.1. Modeling of Porosity

#### 2.1.1. Prediction of Melt Pool Geometry

In the present work, two types of porosity, which are most observed in SLM-processed layers, are considered for calculating the total porosity. The first is lack-of-fusion (LOF) porosity, which arises from insufficient heat input during the melting process and typically forms between adjacent melt pools in both the build and lateral directions. The second is keyhole porosity, caused by vapor depression within the melt pool. Accordingly, accurate modeling of these porosities requires first determining the melt pool and its dimensions.

A critical step in modeling the melt pool during the SLM process is determining the temperature distribution induced by the laser heat source across the powder bed. Throughout the printing process, from the first layer and hatch to subsequent layers and hatches, the temperature field over the powder, substrate, and resulting melt pool is neither constant nor uniform. However, after a short transient period, the system stabilizes and reaches a steady-state condition. Studies by Mukherjee et al. [13,18,19] on the LPBF of various materials including stainless steel 316L, Ti6Al4V, AlSi10Mg, and Inconel 718 have shown that the temperature field and corresponding melt pool volume can vary due to heat transfer between the actively molten pool, the substrate, the surrounding powder, and previously deposited layers. Once this transient phase concludes, a steady-state condition is established, and the melt pool geometry stabilizes. They further reported that porosity calculations should be based on the melt pool shape under this steady-state condition.

Importantly, the transient phase is very brief, and steady-state conditions are typically achieved after the second hatch and layer. Beyond this point, the temperature field and melt pool volume remain essentially unchanged. Consequently, after the second layer and hatch, the melt pool shape and size become effectively uniform [13]. This behavior is observed regardless of the total heat input, as the melt pool volume eventually stabilizes over time.

Based on these findings, porosity modeling in this study assumes that the melt pool attains a uniform and constant shape after the second hatch, following the steady-state condition described by Mukherjee [13]. For the steady-state scenario, Rosenthal’s equation (Equation (1)) [20] can be used to estimate the approximate temperature distribution for a single track. The equation has since been modified and widely accepted to account for the influence of previously deposited adjacent tracks on the temperature distribution and melt pool size of subsequent tracks [20]. Considering that a steady state is reached by the second hatch, Rosenthal’s equation is accordingly adapted, as shown in Equation (2).

(1) $$T=\frac{P\eta }{2\pi kr}exp\left[-\frac{V\left(r+x\right)}{2\left(\frac{k}{\rho C_{p}}\right)}\right]+T_{0}$$

(2) $$T_{n}\left(x,y,z\right)=T_{0}+\sum_{i=1}^{n}\frac{P\eta }{2\pi K\left(T\right)r(i)}exp\left[-\frac{V\left(r(i)+x(i)\right)}{2\left(\frac{K\left(T\right)}{\rho \left(T\right)C_{P}^{m}\left(T\right)}\right)}\right]$$

where P is the laser power, η is the absorptivity coefficient, V is the scanning speed, k is the thermal conductivity, $C_{p}$ is the specific heat capacity of the material, ρ is the density, and r is the distance from the laser heat source $r=\sqrt{x^{2}+y^{2}+z^{2}}$.

Here, P denotes the laser power, η is the absorptivity coefficient, and V represents the scanning speed. The temperature field is also influenced by the material’s physical properties, such as density, thermal conductivity, and specific heat capacity. Since these properties are temperature-dependent, the terms K(T), ρ(T), and $C_{p}^{m}(T)$ represent the temperature-dependent thermal conductivity, density, and modified heat capacity, respectively. These temperature-dependent values are determined using the following relationship [15]. The temperature-dependent material properties can be found in Table 2.

(3) $$D\left(T\right)=\frac{K(T)}{\rho \left(T\right)C_{P}^{m}\left(T\right)}$$

(4) $$C_{P}^{m}\left(T\right)=C_{P}\left(T\right)+L_{f}\frac{\partial f}{\partial T}$$

(5) $$f=\left\{\begin{array}{c} 0\;\;\;\;\;\;T<T_{s}\\ \frac{T-T_{S}}{T_{L}-T_{S}}\;\;\;T_{s}<T<T_{L}\;\\ 1\;\;\;\;\;\;T>T_{L} \end{array}\right.$$

In addition, in Equation (2), the radial distance and corresponding coordinates are modified as follows:

(6) $$\left\{\begin{array}{c} r\left(i\right)=\sqrt{{x\left(i\right)}^{2}+{y\left(i\right)}^{2}+{z\left(i\right)}^{2}}\\ x\left(i\right)=x_{n}+\left(n-i\right)\left(L+Vt_{s}\right)\\ y\left(i\right)=y_{n}+\left(n-i\right)h\\ z\left(i\right)=z_{n} \end{array}\right.$$

In this context, i represents the index of the scanning track and n denotes the total number of scanning tracks, which is set to 2 here, as steady-state conditions are reached after the second track [20]. L corresponds to the length of a single scanning track, $t_{s}$ is the scanning duration for that track, and h indicates the hatch spacing.

To improve the accuracy of temperature field predictions, the effect of heat loss at the part boundaries must be taken into account. Accordingly, multiple heat sink methods are employed to capture this effect [12]. During the LPBF process, heat loss occurs primarily through convection and radiation from the top layer of the melt pool, as well as conduction through the bottom surface and the sides of the part. The temperature loss associated with each heat sink, due to these mechanisms, can be calculated using the following formula:

(7) $$T_{Loss}=T_{0}+\frac{A}{4\pi KR}\left[h\left(T-T_{0}\right)+\epsilon \varrho \left(T^{4}-T_{0}^{4}\right)\right]$$

Here, h represents the convective heat transfer coefficient, A is the area of the corresponding heat sink, ϵ is the emissivity, σ is the Stefan Boltzmann constant, and R is the distance from the calculation point to the heat sink center.

Accordingly, the general equation for the temperature field, which accounts for the temperature rise due to adjacent melt pool formation and the heat losses from convection, conduction, and radiation, is expressed as follows:

(8) $$T_{nm}\left(x,y,z\right)=\sum_{i=1}^{n}\sum_{j=1}^{m}\begin{array}{c} \frac{P\eta }{2\pi K\left(T\right)r\left(i\right)}exp\left[-\frac{V\left(r\left(i\right)+x\left(i\right)\right)}{2\left(\frac{K\left(T\right)}{\rho \left(T\right)C_{P}^{m}\left(T\right)}\right)}\right]\\ -\frac{A(j)}{4\pi KR(j)}\left[h\left(T-T_{0}\right)+\epsilon \varrho \left(T^{4}-T_{0}^{4}\right)\right] \end{array}$$

#### 2.1.2. Prediction of Keyhole Porosity

Bubble formation within the molten pool and the subsequent entrapment of bubbles by the solidification front are considered the two main stages in keyhole porosity formation [16]. The collapse of the melt pool may occur due to vibrations during melt pool formation or variations in scan speed. However, in the present work, the effect of scan speed on bubble collapse has been neglected.

The keyhole porosity ($P_{kh}$) is determined as the ratio of the instantaneous bubble volume generated during melt pool formation ($V_{\mathrm{bubble}}$) to the instantaneous molten volume scanned by the melt pool ($V_{\mathrm{scanned}}$) [16].

(9) $$P_{KH}=p\frac{V_{bubble}}{V_{scanned}}$$

where *p* is the bubble trapping probability; also, *V_bubble_* and *V_scanned_* are calculated using the following equations.

(10) $$\left\{\begin{array}{c} V_{bubble}=n\frac{4}{3}\pi R^{3}dt\\ V_{scanned}=VAdt \end{array}\right.$$

where n is the bubble formation frequency, R is the bubble radius (which can be empirically determined through a calibration procedure described in Section 4), and V is the scan speed. Also, *A* is the instantaneous cross section area scanned by melt pool while it moves in the direction of scan velocity.

According to the keyhole (KH) formation criterion described above, and based on our experimental conditions, KH formation occurs under high line energy regimes—specifically when the scan velocity is low and the laser power is high. Experimental observations indicate that KH formation is likely when the laser power is 250 W and the scan speed is 0.3 m/s. Under these conditions, the melt pool oscillation frequency for stainless steel 316 is approximately 4 kHz [21].

It is noteworthy that the vibration frequency of the melt pool varies depending on the melt pool size and volume, which are governed by the mass of the molten material. In general, as the melt pool size increases, the vibration frequency decreases, while the melt pool depth and cross-sectional area also change accordingly. Caprio et al. [21] established a correlation between the melt pool frequency and its geometrical characteristics, namely depth (*d*) and cross-sectional area (*A*_cs_), as expressed in Equation (11).

(11) $$n=\frac{1.36\times 10^{6}}{\sqrt{A_{cs}}}\;R_{adj}^{2}=86.9\%$$

However, in the present work, we need this value to calculate the KH-induced porosity that only occurs at a high-energy regime (P = 250 W and V = 0.3 m/s) that meets the criterion of KH formation. Accordingly, not only does the 4 kHz of vibration frequency of the melt pool satisfy Equation (11), but this value is derived from experimental results of Capiro et al. [21].

**Figure 1:**
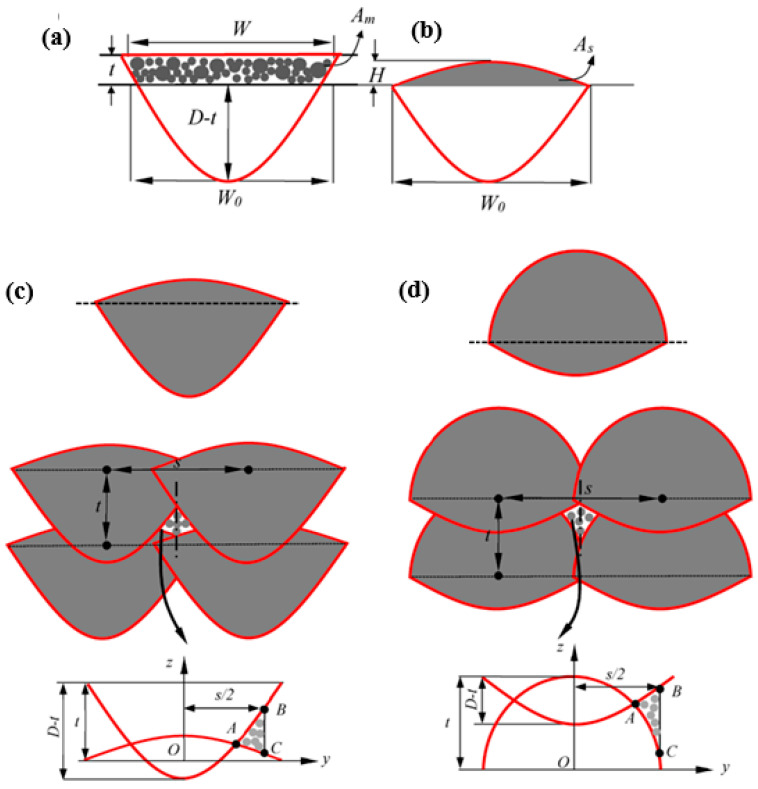
Formation of melt pool (**a**) during printing process in melting stage (**b**) after solidification and formation of cap corresponds to melt pool height. (**c**) Shape of melt pool when *D/t* > 3 and corresponding porosity. (**d**) Shape of melt pool when the 2 < *D/t* < 3 and corresponding porosity.

As reported in previous studies, the cross section of the melt pool has a parabolic shape where the highest width and height of this parabola are equal to the width and depth of the melt pool, as shown in Figure 1c [22]. Accordingly, the equation of this parabola is $y=\frac{4D}{W^{2}}x^{2}$. Considering this parabolic shape, the corresponding area can be calculated by the difference between the rectangle bound by the melt pool width and depth and the area under the parabola, which can be calculated by two times the integral of y over x with upper and lower boundaries of zero and half of the melt pool width, respectively. Following this calculation, the area of melt pool is $A=\frac{2DW}{3}$.

The pores observed in the solidified material result from bubbles being trapped by the solidification front before they can escape from the molten pool’s free surface [16]. To estimate the probability of bubble entrapment, several assumptions are made. First, it is assumed that the bubbles move within the melt pool at the same velocity as the fluid in the build direction, denoted as $v_{f}$. Second, it is assumed that the bubbles originate at the depth of the vapor depression. Under these assumptions, the escape time required for the bubbles to reach the molten pool surface can be calculated as follows [16]:

(12) $$t_{e}=\frac{d}{v_{f}}$$

During solidification, the volume scanned by the melt pool during the bubble escape time is given by *VAt_e_*. Consequently, bubbles that cannot escape from the melt pool result in the formation of porosity. Therefore, the probability of bubble entrapment, denoted as p in Equation (7), can be defined as follows:

(13) $$p=\frac{VA\frac{d}{v_{f}}}{V_{m}},\;V_{m}=\frac{\pi WLD}{6}$$

where *V_m_* represents the volume of the melt pool [22].

By substitution of Equations (10) and (13) in Equation (9), the keyhole porosity is obtained by following equation.

(14) $$P_{KH}=\frac{8nR^{3}d}{WLDv_{f}}$$

It should be noted that keyhole porosity is usually formed in printed material when the heat input is excessive, i.e., high laser power and low scan velocity. However, there is a process combination where the probability of formation of this type of porosity is minimized.

#### 2.1.3. Prediction of Lack-of-Fusion Porosity

Pores due to lack-of-fusion (LOF) represent another type of porosity that forms when the heat input is insufficient, or when the layer thickness and hatch spacing are too large relative to the melt pool dimensions. In contrast to keyhole porosity, which occurs within a single melt pool, LOF forms between multiple melt pools in both the build and hatch directions. Therefore, analyzing LOF requires considering the interaction between melt pool size, hatch spacing, and layer thickness as shown in Figure 1.

To calculate LOF porosity, the shape of the melt pool after solidification and its repetition in the yz plane (representing the build and hatch directions) must be considered. Observations from experiments and the prior literature [22] indicate that, during solidification, a cap forms on top of the melt pool, typically shaped as a semi-ellipse, as illustrated in Figure 1b. According to the law of mass conservation, the mass of the powder in the melted region equals the mass of the solidified melt pool cap. However, because the powder bed contains inherent porosity, a correction factor representing the packed powder density, denoted as ξ and dependent on the powder size distribution, should be included for more accurate predictions. Accordingly, the mass conservation can be expressed as follows:

(15) $$\xi \rho_{melt}A_{m}=\rho_{solid}A_{s}$$

Considering the elliptical area of the cap and the paraboloid shape of the melt pool in the cross section, the following equation can be derived to calculate the height of the solidified melt pool:

(16) $$H=\frac{4}{\pi W_{0}}\xi \left(\frac{\rho_{melt}}{\rho_{soild}}\right)\left[W_{0}t+\frac{1}{12}\left(W^{3}-W_{0}^{3}\right)-D\left(W-W_{0}\right)\right]$$

where H is the cap height, hereafter referred to as the melt pool height, and W and D are the melt pool width during melting and the melt pool depth, respectively, both determined by setting the isothermal contours equal to the material’s melting temperature, also, $W_{0}$ is the width of the melt pool after solidification (Figure 1c) and is given by $W_{0}=2\sqrt{D-t}$, where t is the layer thickness. Accordingly, the paraboloid representing the lower part of the melt pool and the semi-ellipse forming the upper cap are expressed in Equations (17) and (18), respectively.

(17) $$z=\frac{4\left(D-t\right)}{W_{0}^{2}}y^{2}$$

(18) $$z=\frac{H}{W_{0}}\sqrt{W_{0}^{2}-y^{2}}$$

The shape of the melt pool varies depending on the interaction between melt pool depth and layer thickness, as illustrated in Figure 1c,d. When the melt pool depth is significantly greater than the layer thickness, the solidified melt pool takes the form shown in Figure 1c, with a lower part shaped as an upward-bounded paraboloid elongated in the depth direction and an upper part forming a horizontal semi-ellipse. However, with insufficient heat input, the melt pool depth decreases and approaches the layer thickness, resulting in a lower part that is wide and shallow and an upper part that is a more vertical semi-ellipse. The evolution of the melt pool shape as a function of the melt pool depth-to-layer thickness ratio is shown in Figure 1c,d. It should be noted that, if the melt pool depth is equal to or smaller than the layer thickness, proper bonding with the subsequent layer does not occur, leading to rejection of the sample.

To quantify lack-of-fusion (LOF) porosity, the area of material that remains unmolten must be determined. Accordingly, two melt pools in the build directions (Figure 1c,d) were considered based on the layer thickness and hatch spacing. The LOF region for two situations based on the layer thickness and its interaction with melt pool depth are shown in Figure 1c,d. It can be observed that, regardless of the interaction between layer thickness and melt pool depth, which affects the melt pool shape, the LOF region exhibits a symmetrical shape relative to an axis located at half the hatch spacing from the origin of the first melt pool. Consequently, the reference area for calculating LOF is a rectangle with a length equal to the layer thickness and a width equal to half the hatch spacing, giving a reference area of s⋅t/2. As shown in Figure 1c,d, the LOF porosity within this predefined region is calculated as the ratio of the area of triangle ABC to the reference area.

The area of region ABC can be calculated as the difference between the integrals of the paraboloid and the semi-ellipse from point A to point C. As shown in Figure 1c,d, the y-coordinate of point *C* corresponds to half the hatch spacing, i.e., $y_{B}=y_{C}=s/2$. The position of point B, relative to the origin of the two curves (paraboloid and semi-ellipse) at point O, can be determined by solving the following equation using an implicit approach.

(19) $$\frac{4\left(D-t\right)}{W_{0}^{2}}y_{A}^{2}-\frac{H}{W_{0}}\sqrt{W_{0}^{2}-y_{A}^{2}}-D+2t=0$$

Once the coordinates of point *A* are determined, the LOF area can be calculated using the following formula:

(20) $$LOF=\frac{\int_{y_{A}}^{\frac{s}{2}}\left(\frac{4\left(D-t\right)}{W_{0}^{2}}y^{2}-D+2t\right)dy-\int_{y_{A}}^{\frac{s}{2}}\left(\frac{H}{W_{0}}\sqrt{W_{0}^{2}-y^{2}}\right)dy}{\frac{st}{2}}$$

Once the LOF area is determined, the porosity due to lack-of-fusion can be calculated using the relationship proposed by Tang et al. [14] and Ning et al. [12], as follows:

(21) $$P_{LOF}=\left(1-\xi \right)LOF$$

The total amount of porosity can be obtained through the summation of Equations (14) and (21).

(22) $$P_{Total}=P_{LOF}+P_{KH}$$

## 3. Experimental Work

Block-shaped samples (20 × 20 × 40 mm^3^) of stainless steel 316L were fabricated via selective laser melting (SLM) using a Renishaw AM 400 machine manufactured in Gloucestershire, UK equipped with a YFL continuous-wave Ytterbium fiber laser (wavelength 1070 nm, beam diameter 70 μm), capable of a maximum power of 400 W and a maximum scanning speed of 2000 mm/s. During the printing, the fusion region is protected by Argon to avoid oxidation of the material. It ensures that the obtained porosity will be due to phenomena other than oxidation.

The 316L stainless steel powder with the chemical composition presented in Table 3, supplied by 3D Systems, had a particle size distribution ranging from 15 μm to 45 μm, following a Gaussian distribution.

Based on the developed model and our preliminary studies, the most influential factors affecting porosity were identified as laser power, scan speed, layer thickness, and hatch spacing. Accordingly, nine experiments were conducted using the Taguchi L9 design to validate the modeling results, considering different combinations of these process parameters at three levels. To analyze the robustness and repeatability of the results, three samples from each experiment were printed and their porosity was measured. Table 4 presents the experimental design matrix together with the porosity values of each replication.

**Figure 2:**
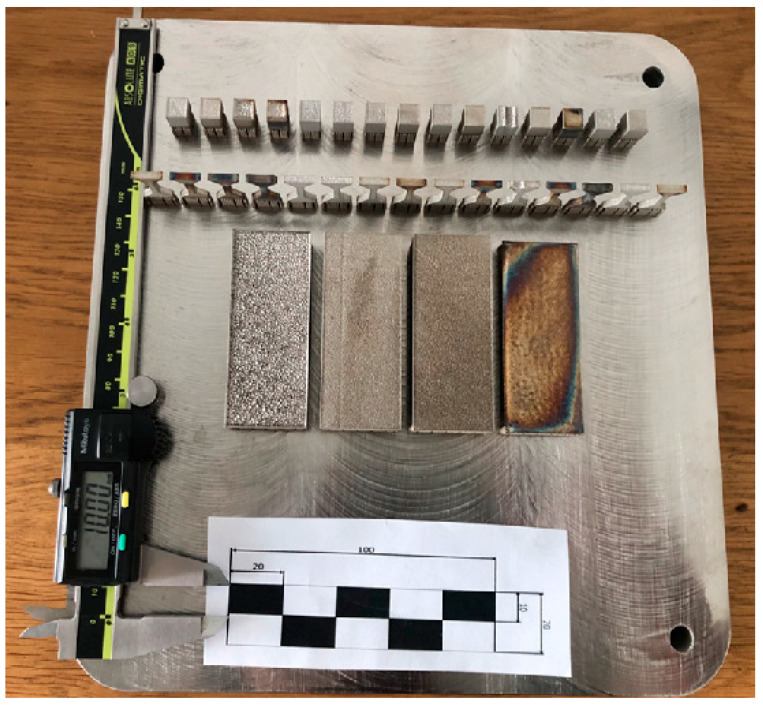
Three-dimensional printed samples.

Porosity measurements were performed using a v|tome|x L-450 industrial micro-CT system. The system provides 3D tomography with a spatial resolution of approximately 2 µm, enabling precise detection and quantification of internal defects. The setup allows high-accuracy reconstruction and visualization of the sample interior, including reliable measurement of pore size and distribution. Porosity measurements were conducted from the interior of the block-shaped samples. Figure 2 shows the block-shaped printed samples together with other samples which are used for parallel research.

The experimental design, along with the calculated volumetric energy density (VED) as the ratio of laser power to the product of scan velocity, layer thickness, and hatch spacing, $VED\;(J/{\mathrm{mm}}^{3})=\frac{P\;(W)}{v\;(mm/s)\;t\;(mm)\;s\;(mm)}$, and the measured porosity values, are provided in Table 4.

## 4. Calibration and Sensitivity Analysis

### 4.1. Calibration of Bubble Radius

In the present work, calibration was performed to develop an equation for predicting the average bubble radius (Equation (10)) as a function of key process parameters, namely laser power and scan speed, using porosity measurements from near-surface layers. To achieve this, μ-CT analysis was conducted to a depth of 20 μm perpendicular to the build direction, allowing observation of porosity at this level.

The 20 μm depth was selected to ensure that no lack-of-fusion (LOF) voids were present, as this corresponds to approximately half of the nominal layer thickness (50 μm). Under the process parameters employed, the melt pool depth was observed to be significantly greater than the layer thickness, confirming that the molten region fully penetrated the previously deposited layer. Consequently, LOF defects typically formed at the boundaries between adjacent layers or scan tracks due to insufficient remelting are highly unlikely to occur within this depth range.

In contrast, keyhole-induced porosity generally forms within a single melt pool, originating near the upper region because of excessive laser energy input and unstable vapor depression dynamics. Therefore, by analyzing a depth of 20 μm from the surface, the focus remains on the region most susceptible to keyhole porosity formation, while avoiding contributions from interlayer defects. This approach ensures that the analysis isolates the keyhole mechanism, providing more accurate interpretation of the porosity behavior under the applied high-energy process conditions.

**Figure 3:**
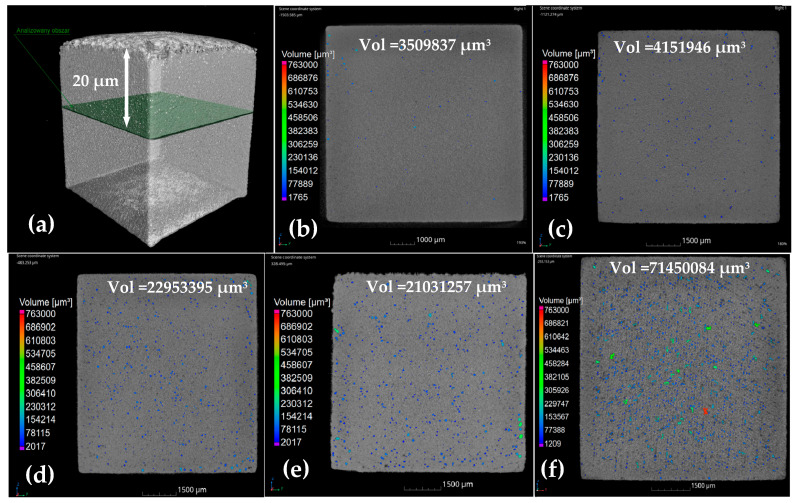
Distribution of porosity (**a**) at the depth of 20 μm from the top surface layer to calibrate the average radius of bubble at constant layer thickness of 50 µm and hatch distance of 110 µm for different processing conditions which is out of our experimental design (**b**) P = 150 W, V = 0.3 m/s (**c**) P = 200 W, V = 0.3 m/s (**d**) P = 250 W, V = 0.3 m/s (**e**) P = 250 W, V = 0.6 m/s (**f**) P = 200 W, V = 0.2 m/s.

The porosity of samples produced under seven different processing conditions obtained by varying laser power and scan speed, as shown in Figure 3, was quantified using μ-CT analysis. The average bubble radius was subsequently correlated with these process parameters using a second-order regression model, expressed as follows:

(23) $$r_{ave}=29.5-0.055P+0.2v_{s}+2.2v_{s}^{2}+0.014Pv_{s}$$

In the study by Wang and Liang [16], a similar correlation was established between the average bubble radius and the normalized enthalpy, as defined in Equation (23). In their work, however, the normalized enthalpy was expressed as a function of scan speed only, while keeping the laser power constant. As presented in Equation (23), both laser power and scan speed contribute differently to the normalized enthalpy, appearing in the numerator and denominator, respectively:

(24) $$E_{n}=\frac{P\eta }{\pi T_{m}\sqrt{k\rho C_{p}Vr^{3}}}$$

Although this enthalpy-based approach was found to yield a strong correlation for Ti6Al4V, its applicability appears to be material-dependent. In the present study on stainless steel 316L, when both laser power and scan speed were simultaneously varied, correlating the average bubble radius solely to normalized enthalpy resulted in a relatively weak correlation coefficient (R^2^ = 0.64). Conversely, directly correlating the bubble size to laser power and scan speed produced a substantially higher correlation (R^2^ = 0.88), suggesting a more reliable predictive relationship for this alloy system. This observation indicates that the bubble formation mechanism and its dependence on thermal history differ between materials, emphasizing the necessity of developing material-specific empirical models. It is also worth noting that both the enthalpy-based and parameter-based approaches are empirical in nature; however, for stainless steel 316L, the latter provides superior predictive accuracy and better reflects the combined influence of laser power and scan speed on porosity evolution.

The keyhole formation is a type of porosity that cannot be studied by considering a single factor such as scan speed or laser power alone. To effectively analyze it, both factors must be considered. According to the research carried out by Cunningham et al. [23], synchrotron X-ray imaging under LPBF-relevant conditions revealed systematic variations in the shape of the keyhole depression in titanium. The keyhole generally exhibits a J-shaped morphology, with a distinct tail behind the depression at high laser power and low-to-medium scan velocities. At higher velocities, this tail becomes less visible, and the opening widens. The primary difference in keyhole geometry across various laser power–velocity combinations lie in the extent of this opening. At high power and lower velocities, the strong drilling effect leads to a deep, narrow keyhole, where enhanced multiple reflections increase energy absorption and reinforce the J-shaped form. In contrast, at higher scan velocities (lower energy densities), the reduced penetration rate results in a shallower keyhole with a larger opening, as the laser beam exits after fewer reflections and recoil pressure acts near the keyhole mouth.

### 4.2. Sensitivity Analysis of Coefficients and Constants

To evaluate the robustness of the proposed porosity model and justify the simplifying assumptions, a sensitivity analysis was conducted on key constants or coefficients that can be directly modified in the model. Specifically, the analysis considered laser absorptivity (*η*), the coefficient of thermal expansion (constant vs. temperature-dependent), and the effect of heat loss due to convection in the melt pool. The analysis was performed by calculating the mean absolute percentage of error (MAPE) between the predicted and measured porosity across the nine experiments in the revised validation dataset (Table 4) using the following equation.

(25) $$MAPE=\frac{1}{N}\sum_{i=1}^{N}\frac{\left|{Porosity}_{Measured}-{Porosity}_{predicted}\right|}{{Porosity}_{Measured}}\times 100$$

**Figure 4:**
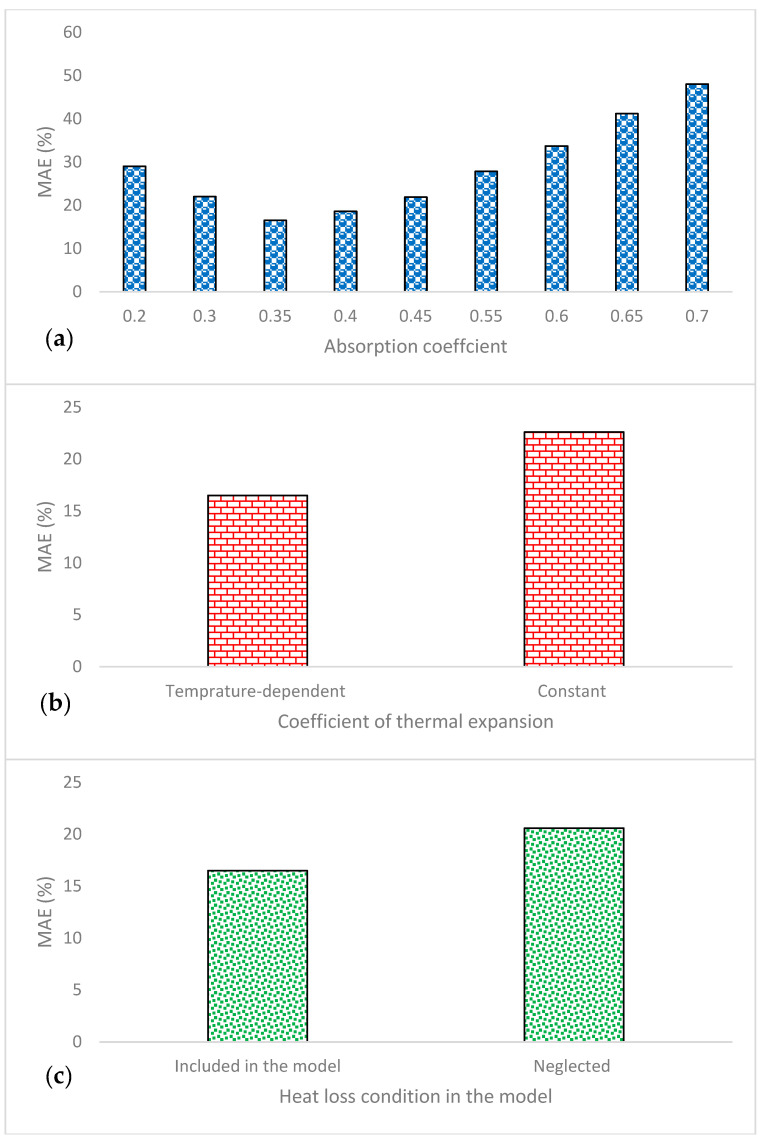
Effect of key constants and coefficients on porosity predictions: (**a**) laser absorptivity (η), (**b**) coefficient of thermal expansion, (**c**) convective heat loss.

For laser absorptivity (*η*), values were varied from 0.2 to 0.7 in increments of 0.05. The MAE reached a minimum value of 16.0% when *η* = 0.35, as shown in Figure 4a, indicating the optimal absorptivity for the current model. Regarding the coefficient of thermal expansion, the comparison between a constant and a temperature-dependent formulation revealed improved prediction accuracy when the temperature-dependent coefficient was used (Figure 4b). Similarly, including convective heat loss in the melt pool further improved accuracy, as illustrated in Figure 4c. These results confirm that these constants and coefficients have a measurable impact on model predictions and justify their inclusion in the formulation.

Viscosity (*μ*) and surface tension (*γ*) are not explicitly included in the current porosity model. They are reported only in the manuscript to provide context for keyhole formation criteria, which are discussed based on other literature formulations. Their values do not directly affect the porosity predictions in this study. Accordingly, these parameters were not included in our sensitivity analysis.

## 5. Results and Discussion

### 5.1. Paramertric Influence

To design a process window including the impact of process factors, showing regions where there is influence of process parameters, namely laser power, scan speed, hatch distance, and layer thickness, on porosity formation, it is first necessary to consider the probability of porosity occurrence. Accordingly, based on the study reported by Liu et al. [6], certain criteria defined through non-dimensional numbers are introduced to determine whether lack-of-fusion (LOF) or keyhole (KH) porosity occurs. These are denoted as π_1_ and π_2_, respectively, and are calculated using the following formulas:

(26) $$\pi_{1}=\frac{\pi DW}{2ht}$$

(27) $$\pi_{2}=\frac{\gamma D}{v_{x}\mu \sigma }$$

(28) $$v_{x}=\frac{DWv^{2}}{4\left(\frac{k}{\rho C_{p}}\right)\left[\frac{3}{2}\left(d+\frac{W}{2}\right)-\sqrt{\frac{DW}{2}}\right]}$$

where *D* is the melt pool depth, *W* is the melt pool width, *s* is the hatch distance, t is the layer thickness, *γ* is the surface tension, *vₓ* is the melt backflow speed, *σ* is the laser spot radius, and *μ* is the dynamic viscosity of the material. The above-mentioned properties, considering stainless steel 316L as the printing material, are provided in Table 2.

**Figure 5:**
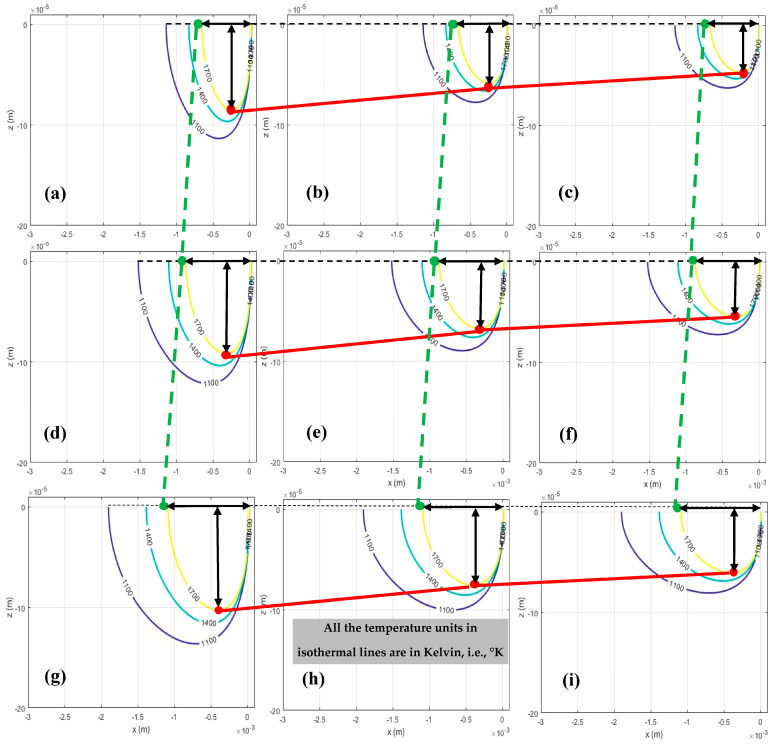
Side, i.e., xz views of melt pool in melting stage under different laser powers and scan speeds: (**a**) P = 150 w, v = 0.2 m/s; (**b**) P = 150 w, v = 0.6 m/s; (**c**) P = 150 w, v = 1 m/s; (**d**) P = 200 w, v = 0.2 m/s; (**e**) P = 200 w, v = 0.62 m/s; (**f**) P = 200 w, v = 1 m/s; (**g**) P = 250 w, v = 0.2 m/s; (**h**) P = 250 w, v = 0.6 m/s; (**i**) P = 250 w, v = 1 m/s.

**Figure 6:**
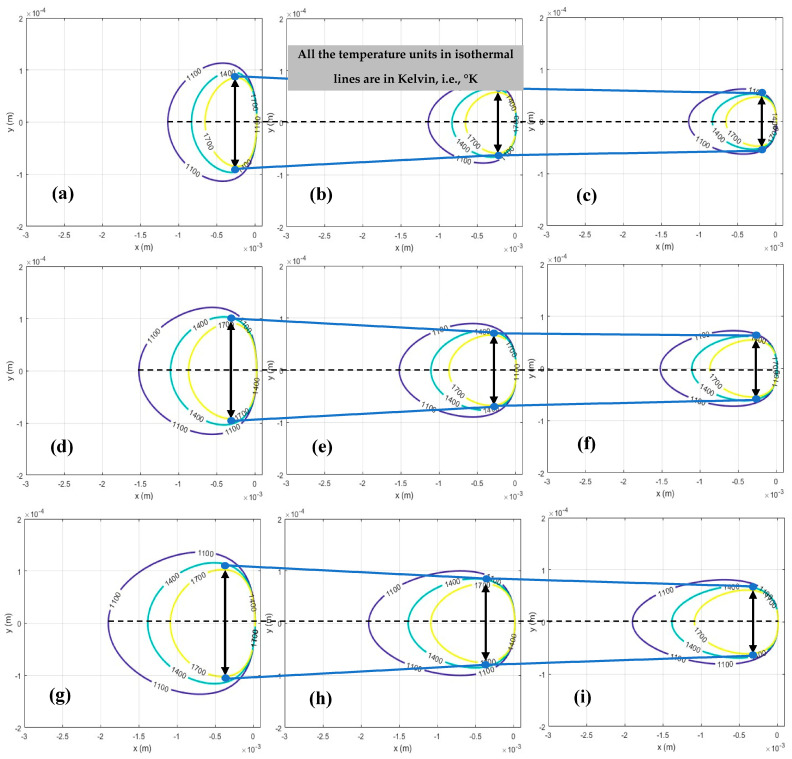
Top, i.e., yx views of melt pool in melting stage under different laser powers and scan speeds: (**a**) P = 150 w, v = 0.3 m/s; (**b**) P = 150 w, v = 0.65 m/s; (**c**) P = 150 w, v = 1 m/s; (**d**) P = 200 w, v = 0.3 m/s; (**e**) P = 200 w, v = 0.65 m/s; (**f**) P = 200 w, v = 1 m/s; (**g**) P = 250 w, v = 0.3 m/s; (**h**) P = 250 w, v = 0.65 m/s; (**i**) P = 250 w, v = 1 m/s.

**Figure 7:**
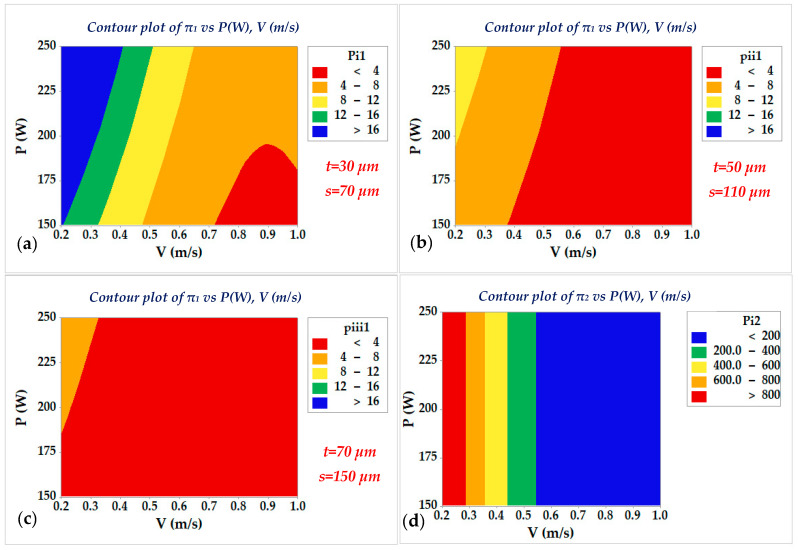
Variation in π_1_ with respect to (**a**–**c**) laser power and scan speed under different combinations of layer thickness and hatch spacing. (**d**) Variation in π_2_ with respect to laser power and scan speed.

According to the defined criteria, LOF formation occurs when π_1_ is less than 4, whereas KH porosity becomes highly probable when π_2_ exceeds 800. Therefore, prior to analyzing the effects of process parameters, the melt pool depth and width, along with the corresponding π_1_ and π_2_ values, were calculated to define the boundaries for porosity formation. The results showing the variations in melt pool geometry and the evolution of π_1_ and π_2_ are presented in Figure 5, Figure 6 and Figure 7.

According to the results presented in Figure 5 and Figure 6, which were derived in MATLAB R2023b software, it is observed that increasing the laser power and decreasing the scan speed led to an increase in both melt pool depth and width, that is attributed to a distribution of temperature at the melting point to a wider region over the powder bed. Consequently, this affects the values of π_1_ while they are simultaneously considered with the layer thickness and hatch spacing (shown in Figure 7a) that are related to the formation of LOF. Also, it impacts the π_2_ (as shown in Figure 7b), which is associated with the formation of KH.

The contour plots of the criteria π_1_ and π_2_, which were obtained using the MINTAB 18 software, have been presented in Figure 7. According to Figure 7a, it is seen that, for a hold value of layer thickness and a hatch spacing of 30 µm and 70, respectively, the red region that is bounded by the scan speed range of 0.75 m/s to 1 m/s and laser power range of 150 W to 180 W results in the formation of LOF, since the corresponding π_1_ value adopts values less than 4. When the hold values of layer thickness and hatch spacing increase to 50 µm and 110 µm, it is seen that area under the red color expands, implying that LOF occurs at wider ranges of scan speed and laser power, whereas, if the scan speed is more than 0.5 m/s, the LOF occurs at all the levels of laser power. Figure 7c also shows that, for the combination of a layer thickness of 70 µm and a hatch space of 150 µm, the LOF-free region is limited to the range where the laser power is greater than 180 W and the scan speed is less than 0.3 m/s.

Also, Figure 7d shows that, irrespective of the values of laser power, the red region which represents the occurrence of KH becomes bounded by a scan speed less than 0.3 m/s. Accordingly, the π_2_ values at any value of laser power exceed 800 when the scan speed is less than 0.3 m/s.

Based on the melt pool dimensions and the π_1_ and π_2_ criteria, the combination of 250 W laser power and 0.4 m/s scan speed yields the porosity-free LPBFed sample at any values of layer thickness (varying between 30 µm and 70 µm) and hatch space (varying between 70 µm and 150 µm). However, this criterion sometimes overestimates the values of π_1_ because of neglecting the formation of the cap at the top of the melt pool due to surface tension during solidification, as described in Section 2. Accordingly, to obtain a more accurate process window, this effect on the formation of LOF should be discussed first. Since LOF typically develops in multi-track and multi-layer configurations, the effects of layer thickness and hatch spacing must also be considered. Therefore, to further analyze LOF formation, laser power and scan speed were fixed at 250 W and 0.4 m/s, respectively (corresponding to the yellow dashed region in Figure 7a), while the effects of hatch distance (70 μm, 110 μm, and 150 μm) and layer thickness (30 μm, 50 μm, and 70 μm) were investigated.

**Figure 8:**
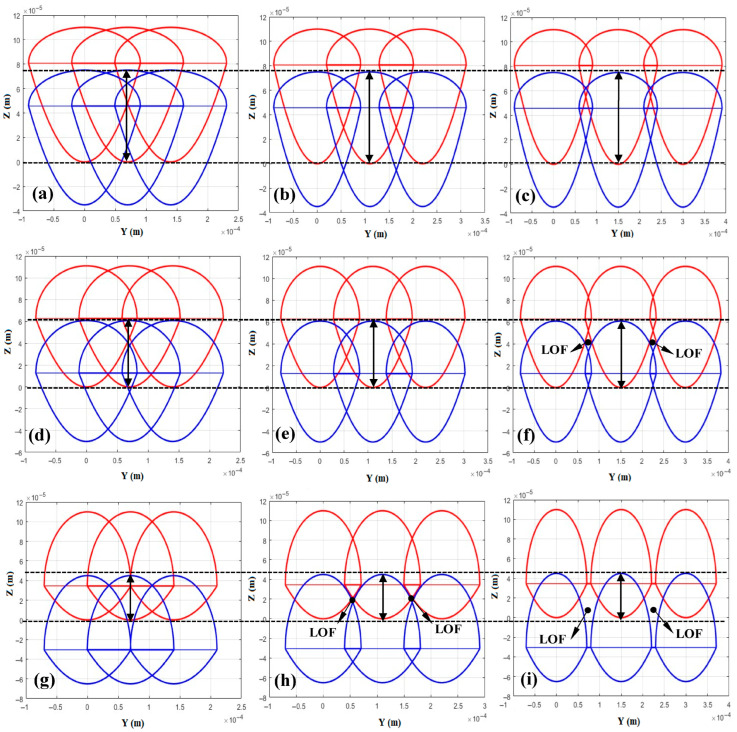
Cross section of two-layer and three-track melt pool configurations under scan speed of 0.3 m/s and laser power of 250 W for different hatch distance and layer thickness conditions: (**a**) s = 70 μm, t = 30 μm; (**b**) s = 110 μm, t = 30 μm; (**c**) s = 150 μm, t = 30 μm; (**d**) s = 70 μm, t = 50 μm; (**e**) s = 110 μm, t = 50 μm; (**f**) s = 150 μm, t = 50 μm; (**g**) s = 70 μm, t = 65 μm; (**h**) s = 110 μm, t = 70 μm; (**i**) s = 150 μm, t = 70 μm.

Figure 8 presents the multi-track and multi-layer cross sections of the melt pools after solidification, obtained from the analytical model considering two layers and three tracks in MATLAB software. The simulations were performed under varying hatch distances and layer thicknesses, while the scan speed and laser power were kept constant at 0.3 m/s and 250 W, respectively. According to the figure, when the layer thickness is 30 μm, the LOF porosity remains negligible, even with increasing hatch distance (Figure 8a–c). A similar trend is observed for a layer thickness of 50 μm, where no noticeable LOF appears up to a hatch distance of 110 μm (Figure 8d,e). However, when the hatch distance increases to 150 μm, LOF porosity becomes significant, as shown in Figure 8f.

Furthermore, when the layer thickness reaches 70 μm, porosity formation begins at a hatch distance of 110 μm, and increasing the hatch distance to 150 μm leads to a drastic rise in LOF. Conversely, for a fixed hatch distance of 150 μm, increasing the layer thickness from 30 μm to 70 μm results in a pronounced increase in LOF formation.

As the hatch distance increases, the spacing between two successive melt pools becomes larger. Under such conditions, when the melt pool width is insufficient to bridge this gap, the formation of LOF becomes unavoidable. This explains why, at lower layer thicknesses, it remains possible to achieve LOF-free samples even with increasing hatch distance, as shown in Figure 8a–c. However, at higher layer thicknesses, increasing the hatch distance leads to the initiation and rapid growth of LOF porosity, as illustrated in Figure 8g–i.

In addition, when the layer thickness increases, if the melt pool depth, which depends on scan speed and laser power, is not sufficiently deep, the solidified pool tends to form a cap with a higher surface curvature. This reduces the effective interlayer bonding length, as indicated by the bold black arrows in Figure 8. This trend can be observed regardless of the hatch distance. Nevertheless, when the hatch distance is relatively large (i.e., 150 μm), increasing the layer thickness results in a substantial rise in porosity, as seen by comparing Figure 8c,f,i.

**Figure 9:**
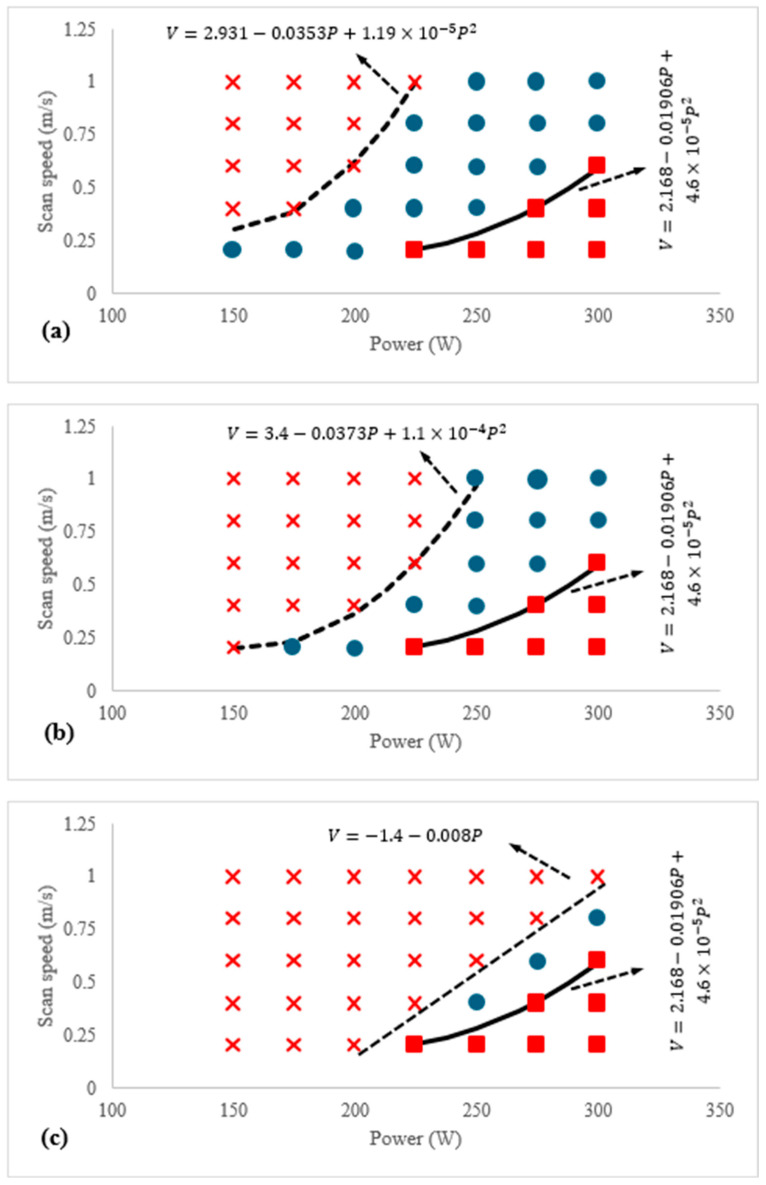
The process window encompasses different levels of laser power and scan speed for (**a**) layer thickness of 30 µm and hatch space of 70 µm, (**b**) layer thickness of 50 µm and hatch space of 110 µm, (**c**) layer thickness of 70 µm and hatch space of 150 µm.

According to the discussions above, a modified process window has been defined in this work to give a more precise understanding and estimation regarding the selection of appropriate process factors for achieving these porosity-free effects. It should be noted that, for constructing this window, samples with porosity below 0.5% were considered sound and are represented by blue circles. The resulting process window is shown in Figure 9. It can be observed that, with increasing hatch spacing and layer thickness, the keyhole region remains almost unchanged, as keyholes form within a single melt pool. This region is located on the right side of the graph and is represented by red-filled squares along with their corresponding boundaries. In contrast, the regions marked with red crosses correspond to the combinations of laser power and scan speed that led to the formation of lack-of-fusion (LOF) defects. A comparison of Figure 9a–c shows that the graph becomes increasingly confined on the left side, indicating that increasing the hatch spacing and layer thickness promotes LOF formation, thereby narrowing the process window for producing sound samples.

### 5.2. Confirmation

The experimental validation was carried out by comparing the values of porosity reported in Table 4 and those predicted by the theoretical framework. To do so, the mean of the three measurements (reported in Table 4), together with the values of the standard deviation and the upper and lower values of the confidence interval (CI), have been calculated and are presented in Table 5. This table also includes the predicted values of porosity together with the point-to-point error, which was obtained as the percentage of the division of the absolute difference in measured and predicted values over the measured value.

**Figure 10:**
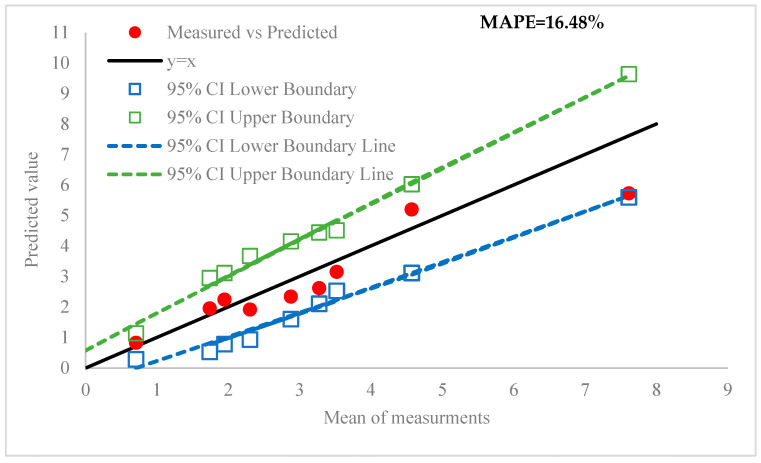
Comparison between the predicted values of porosity and mean of three measurements, including the 95% confidence interval boundaries.

Figure 10 compares the porosity of 3D-printed samples obtained from both experimental measurements and simulation predictions. As shown, there is a strong agreement between the measured and predicted values, with the error percentage ranging from 10.5% to 24.78% and an average error of 16.48%. According to the provided results, it is seen that the predicted results fall within the 95% confidence interval for eight out of nine sets and only a single result sits on the border line. It affirms that the model can predict the porosity with an acceptable prediction error.

Building on these error values, it is important to note that several aspects of the modeling approach may contribute to the observed mismatch between the measured and predicted results. Variations in particle size can lead to differences between the actual and assumed initial powder packing density in the simulations, thereby influencing the final porosity outcomes.

Furthermore, the assumption that the melt pool geometry becomes uniform after the second hatch and second layer, based on previous finite element studies [13], may not fully represent the real process behavior. This can introduce discrepancies in the predicted melt pool characteristics and, consequently, in the estimation of both lack-of-fusion (LOF) and keyhole (KH) porosity.

The potential effect of remelting in the underlying layers, which may slightly modify the geometry of subsequently deposited layers, was also not considered. Although likely small, this effect can still influence the overall porosity, particularly under keyhole mode melting conditions.

Additional uncertainties arise from simplifying assumptions such as constant bubble frequency, uniform melt pool geometry after two hatches, neglecting scan-speed-dependent bubble collapse, and simplified modeling of melt pool convection and Marangoni flow. Despite these simplifications, the prediction errors generally remain within the standard deviation range, either fully inside or close to the upper and lower limits of the shaded region.

## 6. Conclusions

In the present work, an analytical model was developed to correlate LPBF process parameters with porosity formation due to lack-of-fusion (LOF) and keyhole (KH) mechanisms. First, the temperature field was modeled to determine the melt pool dimensions and the vapor generated during melting, which were used to calculate porosity due to keyhole formation. Porosity from LOF was then estimated by considering the overlap of solidified melt pools in multi-track and multi-layer configurations. The predicted porosity values were compared with experimental results for validation. The main findings are summarized as follows:

1. The predicted porosity values showed reasonable consistency with the experimental measurements, with maximum, minimum, and mean prediction errors of 24.78%, 10.5%, and 16.48%, respectively, and generally fell within the 95% confidence interval, consistent with previous studies [16,19].
2. Analysis indicated that increasing the energy density, either by reducing scan speed or increasing laser power, initially reduces LOF porosity. Beyond a certain threshold, further increases in energy density led to higher porosity due to keyhole formation.
3. Increasing layer thickness was found to increase LOF porosity and reduce interlayer bonding. Similarly, increasing hatch distance also raises LOF. However, at high energy densities, the melt pool becomes sufficiently large, and variations in layer thickness or hatch distance have a limited impact on LOF porosity.
4. It should be noted that, since in this work the bubble radius is determined using an empirical approach, our computational framework for porosity prediction can be classified as semi-analytical when the process parameters are designed such that keyhole formation becomes dominant. Nevertheless, the development of a fully predictive model remains an open issue, particularly concerning the modeling and calibration of bubble size and its relationship with the printing parameters.
5. Future work will focus on extending the current process porosity model to include detailed porosity descriptors (size distribution, shape, anisotropy, and tortuosity), aiming to capture the chaotic nature of pore formation and improve the physical interpretability of the model.

## Figures and Tables

**Table 1:** Overview of existing porosity modeling studies in LPBF, outlining key aspects and research gaps.

Reference	Type of Modeled Porosity	Modeling Strategy	Remarks
Wang and Liang [10]	Lack-of-fusion	Analytical	Low prediction accuracy because of neglecting formation of cap over melt pool that significantly impacts the LOF. Lower generalizability because of confirmation of results only by variation in power and scan speed while the impact of layer thickness and hatch spacing were not considered. Lower generalizability at wider range because it only included the impact of lack-of-fusion on porosity. The computational efficiency is same as both models derived from their model through developing a code in MATLAB.
Ning et al. [11]	Lack-of-fusion	Analytical	Neglecting the cap formation in prediction of LOF that causes shift between the measured and modeled value. Lack of generalizability since the model has not been verified over factors such as hatch spacing and layer thickness. The computational cost is somehow same as an analytical model which was coded in MATLAB.
Ning et al. [12]	Lack-of-fusion	Analytical	Neglect of cap formation in the prediction of lack-of-fusion (LOF) defects, leading to a shift between the measured and modeled values. Limited generalizability, as the model was not verified for other process factors such as hatch spacing and layer thickness. Comparable computational cost, since the approach was also based on an analytical model implemented in MATLAB.
Tang et al. [14]	Lack-of-fusion	FEM	The model has high accuracy because of considering all the melt pool features, including cap. It is generalizable as it was checked and verified for different material. The computational efficiency is not high because of using numerical simulation that takes long time to be well implemented.
Wang and Liang [16]	Keyhole	Analytical	The porosity prediction in the model is limited only to high-energy regime that induces keyhole porosity. The model was verified only by variation in scan speed that hinders its generalizability. Computational efficiency can be comparable to this work that both use analytical model in MATLAB.
King et al. [17]	Keyhole	FEM	The process lacks generalizability because it only considers the impact of one single track. They used CFD-based simulation that is time-consuming and hinders the computational efficiency.

**Table 2:** Properties of stainless steel used in the simulation model [15].

Properties	Symbol	Unit	Value
Density of solid	*ρ_s_*	Kg/m^3^	8084 − 0.4209T − 3.894 × 10^−3^T
Density of liquid	*ρ_c_*	Kg/m^3^	7433 − 0.0393T − 1.8 × 10^−2^T
Specific heat of solid	*C_s_*	J/kg°K	462 + 0.134T
Specific heat of liquid	*C_L_*	J/kg°K	775
Thermal conductivity of solid	*K_s_*	W/m°K	9.248 + 0.01571T
Thermal conductivity of liquid	*K_L_*	W/m°K	12.41 + 0.00327T
Melting temperature	*T_m_*	°K	1723
Evaporation temperature	*T_v_*	°K	3090
Viscosity of liquid metal	*µ*	Kg/ms	10^(2358.2/*T* − 3.5958)^
Surface tension	*γ*	Kg/s^2^	1.87
Absorptivity	*η*		0.35

**Table 3:** Chemical composition of stainless steel 316L.

Element	C	Si	Mn	P	S	Cr	Ni	Mo	Fe
wt.%	0.03	0.75	2.0	0.045	0.03	16–18	10–14	2–3	Balance

**Table 4:** Experimental design matrix.

No	P (W)	V (m/s)	t (μm)	s (μm)	VED (J/mm^3^)	Measured Porosity (%)			Predicted Porosity (%)	Error with Respect to Mean Value (%)
						1	2	3		
1	150	0.20	30	70	357.14	0.68	0.89	0.55	0.827	17.1
2	150	0.6	50	110	45.45	4.32	5.24	4.15	5.196	13.7
3	150	1	70	150	14.29	7.44	8.5	6.9	5.726	24.78
4	200	0.20	50	150	133.33	2.25	1.78	2.88	1.916	16.8
5	200	0.6	70	70	68.03	3.16	3.45	3.95	3.15	10.5
6	200	1	30	110	60.61	3.41	2.75	3.66	2.618	20
7	250	0.2	70	110	162.34	1.87	1.52	2.45	2.241	15.2
8	250	0.6	30	150	92.59	1.61	1.33	2.28	1.954	12.3
9	250	1	50	70	71.43	2.95	2.33	3.35	2.34	18.4

**Table 5:** Statistical analysis of measurements.

No	Mean Value (%)	Standard Deviation	Lower CI	Upper CI	Predicted Value (%)	Error (%)
1	0.7067	0.17155	0.2807	1.1326	0.827	17.1
2	4.57	0.58645	3.1141	6.0259	5.196	13.7
3	7.6133	0.81395	5.5926	9.6341	5.726	24.78
4	2.3033	0.55195	0.9331	3.6736	1.916	16.8
5	3.52	0.3996	2.5279	4.5121	3.15	10.5
6	3.2733	0.47015	2.1062	4.4405	2.618	20
7	1.9467	0.4697	0.7805	3.1128	2.241	15.2
8	1.74	0.48815	0.5281	2.9519	1.954	12.3
9	2.8767	0.51395	1.6008	4.1526	2.34	18.4

## Data Availability

The original contributions presented in this study are included in the article. Further inquiries can be directed to the corresponding author.
